# Structural biology of the intrinsic cell death pathway: what do we know and what is missing?

**DOI:** 10.5936/csbj.201204007

**Published:** 2012-04-16

**Authors:** Erinna F. Lee, W. Douglas Fairlie

**Affiliations:** a The Walter and Eliza Hall Institute of Medical Research, Parkville, Victoria 3052, Australia and Department of Medical Biology, The University of Melbourne, Parkville, Victoria 3010, Australia

**Keywords:** Apoptosis, Bcl-2, Bax, BH3 domain, Apaf-1, Apoptosome

## INTRODUCTION

Programmed cell death, or apoptosis, is essential for the proper development and maintenance of tissue homeostasis in metazoans. Cells that are damaged, dangerous or superfluous undergo apoptosis and are removed unobtrusively by professional phagocytes, such as macrophages and dendritic cells [1–3]. This is unlike other forms of cell death such as necrosis, which elicits an inflammatory response due to the release of noxious cellular contents from the dying cell. The apoptotic demise of the cell is executed by a group of proteases, known as caspases (cysteine-dependent asparate-specific proteases), which once activated, cleave vital cellular substrates.

**Figure 1:**
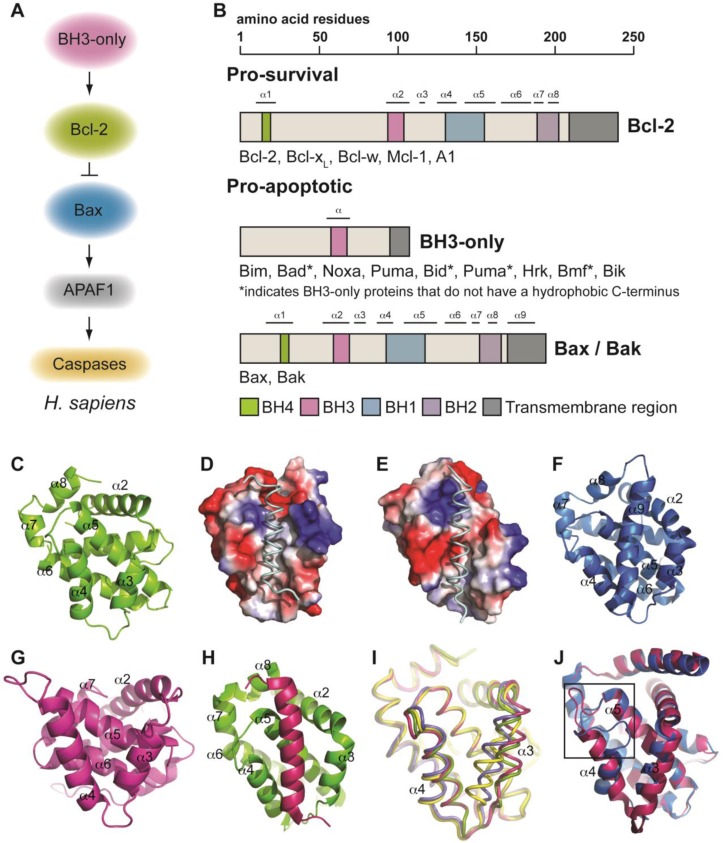
**A)** Schematic of the Bcl-2 regulated cell death in humans. **B)** Domain architecture of the Bcl-2 family of proteins. These proteins are characterized by the presence of up to 4 regions of sequence homology, known as Bcl-2 homology (BH) domains. **C)** The structure of the pro-survival protein, Bcl-x_L_ (PDB: 1MAZ) comprises a helical bundle arrangement that typifies the Bcl-2 protein family fold. Surface electrostatic representation of **D)** Bcl-x_L_ and **E)** Bfl-1 bound to Bim BH3 peptide show different charge distributions in the BH3 domain binding groove (PDB: 3FDL and 2VM6 respectively). **F)** Structure of the multi-domain pro-apoptotic protein, Bax (PDB: 1F16). The structure resembles that of the pro-survival protein and has the C-terminal α9 helix binding in the canonical hydrophobic groove. **G)** The only BH3-only protein, Bid, has a three-dimensional structure (PDB: 2BID) similar to that of the pro-survival and multi-domain pro-apoptotic proteins. Only Bid and possibly Bik are structured BH3-only proteins, the remaining members of this sub-class are thought to be intrinsically unstructured proteins. **H)** The structure of the BH3 domain from Bim (pink) bound to Bcl-x_L_ (green) (PDB: 3FDL) is typical of interactions between BH3 domains from pro-apoptotic proteins and pro-survival proteins. **I)** Overlay of structures of Bcl-xL from complexes of it in its apo form (yellow, PDB: 1PQ0), or bound to ABT-737 (green, PDB: 2XYJ), an ABT-737 analogue (blue, PDB: 3INQ) or Bim BH3 peptide (pink, PDB: 3FDL) highlights significant differences in the arrangement of helices α3 and α4 that line the ligand binding groove. **J)** CED-9 also undergoes significant conformational changes upon binding the BH3 domain from Egl-1. The unstructured loop connecting helices α4-α5 (boxed) moves significantly in the complexed structure (pink, peptide has been removed for clarity, PDB: 1TY4), compared to in apo-CED-9 (blue, PDB: 1OHU).

Upstream of caspase activation, a group of proteins that plays a vital role in the regulation of apoptosis signaling is the Bcl-2 family of proteins. These proteins are defined by the presence of at least one, and up to four, regions of sequence homology known as cl-2 omology (BH) domains and the family comprises three sub-classes, each distinguished by their functional role (Figure 1A, B) [4]. The first of these sub-classes are the pro-survival Bcl-2 proteins (Bcl-2 itself, Bcl-x_L_, Bcl-w, Mcl-1 and Bfl-1) which are responsible for the protection of cells against various death stimuli such as cytokine withdrawal or DNA damage. The second sub-class contain the Bax/Bak proteins. These are multi-BH domain-containing proteins that are closely related to their pro-survival counterparts in their domain architecture. However, in contrast, Bax and Bak promote cell death and are critical mediators of apoptosis as evidenced by the severe phenotype of the *bax*^*-/-*^*bak*^*-/-*^ mice, and the resistance of cells derived from these mice to most forms of stress-induced apoptosis, and the enforced expression of the BH3-only proteins [5]. These BH3-only proteins constitute the third sub-class of Bcl-2 family proteins and are the initiators of the apoptotic cascade. As suggested by their name, BH3-only proteins are related to each other, and to other members of the Bcl-2 family, by the presence of just the BH3 domain. The BH3-only proteins are upregulated transcriptionally and post-translationally upon receipt of a death stimulus.

A number of models have been proposed for how BH3-only proteins activate Bax and Bak. In one model, known as “indirect activation”, Bax and Bak activation is spontaneous and their pro-apoptotic activities are exerted once released from pro-survival protein sequestration. This release occurs following binding of the BH3-only proteins to the pro-survival proteins [6, 7]. An alternate model, known as “direct activation”, suggests that a sub-group of the BH3-only proteins are able to bind directly to, and trigger the activation of Bax and Bak. Here, pro-survival proteins act to sequester these “activator” BH3-only proteins [8–11]. A third model, known as the “embedded together” model proposes that the pro-survival proteins are dominant-negative regulators of Bax and Bak and bind both the BH3-only proteins as well as Bax and Bak in the mitochondrial membrane, thereby inhibiting apoptosis [12, 13]. Very recently, a fourth “unified” model encompassing the major aspects of the above-mentioned models has been proposed [14]. Significantly, this model takes into account the differential efficiencies by which each model of cell death activation occurs. Regardless of the mechanism by which Bax/Bak activation occurs, the key point of no return in the apoptotic cascade is the permeabilization of the outer mitochondrial membrane (MOMP). MOMP occurs when Bax/Bak oligomerize on the outer mitochondrial membrane, forming pore-like structures through which apoptogenic factors such as cytochrome *c* are released into the cytosol from the space between the inner and outer mitochondrial membranes. Cytochrome *c* then binds to an adaptor protein known as Apaf-1, which leads to the formation of an oligomeric assembly known as the “apoptosome”. Formation of the apoptosome leads to the activation of the cellular demolitionists, the caspases and in turn this spells the inevitable death of the cell. It should be noted that alternative pathways independent of the Apaf-1-cytochrome *c* axis have been reported [15].

Given the importance of apoptosis in the maintenance of tissue homeostasis and the removal of rogue cells, it comes as no surprise that dysfunctional apoptosis signaling leads to disease manifestation. Deficient apoptosis leads to an accumulation of unwanted cells and the inability to respond normally to apoptotic stimuli. Diseases such as cancer and autoimmune disorders are characterized by this. In contrast, excessive apoptosis results in diseases in which cells are removed inappropriately. Examples of these include neurodegenerative disorders. The therapeutic targeting of the Bcl-2-regulated apoptotic pathway has therefore been an attractive avenue for the treatment of diseases characterized by such defects. As one would expect, a detailed molecular understanding of how these proteins interact functionally and structurally is essential to the development of safe and effective drugs.

Since the initial discovery of the *bcl-2* gene [16] and the subsequent characterization of the function of its gene product, Bcl-2 [17], more than 20 years ago, our understanding of how these proteins work together to regulate apoptosis has expanded enormously. In addition, a large body of structural studies has been undertaken that have provided us with a “family album” of the various components that make up the Bcl-2-regulated apoptotic pathway. Here, we will review the structural information available to us in this area, as well as discuss where structural information is still lacking.

## STRUCTURAL BIOLOGY OF THE BCL-2 PROTEINS

### The pro-survival Bcl-2 proteins

The first atomic resolution structure of a Bcl-2 family member was that of the mammalian pro-survival protein Bcl-x_L_ [18] (Figure 1C). The structure revealed an eight helical bundle in which the α-helices are connected by loops of varying lengths. Two central helices, α5 and α6, form the core of the protein and are primarily hydrophobic in nature. These are sandwiched on one side by the α1 and α2 helices, and on the other side by α3 and α4. Two more helices, α7 and α8, complete the structure. The BH1, BH2 and BH3 domains are not contiguous in the primary sequence, however, in the three-dimensional fold, these conserved domains form a surface exposed hydrophobic groove. This groove is formed from helices α2- α5 and α8 and is the binding site for BH3 domains from the pro-apoptotic proteins (discussed below).

Since then, structures of three more of the five mammalian pro-survival proteins in their unliganded states have been determined (Bcl-2, Bcl-w, and Mcl-1) and reveal that the structure of Bcl-x_L_, typifies a Bcl-2 protein fold [19–22]. It should be noted that all structures of pro-survival proteins have been solved utilizing C-terminally-truncated recombinant proteins. Structures of truncated pro-survival Bcl-2 proteins from the nematode *C. elegans* (CED-9) [23], and the schistosome *S. japonicum* (sjA) [24], have also been solved, and demonstrate the conservation of the fold through evolution. The closest to a full-length structure is that of Bcl-w [25]. Here, an additional helix, α9, binds into its own hydrophobic groove and gets displaced upon ligand binding [26]. Close inspection and comparison of the four structures reveals differences in the nature of the different hydrophobic grooves. These include, the “openness” of the groove and their electrostatic character (Figure 1D, E). These idiosyncrasies could potentially contribute to the selectivity differences observed in pro-survival protein:pro-apoptotic protein interactions.

### The pro-apoptotic Bax/Bak proteins

The Bax/Bak proteins are multi-domain pro-apoptotic proteins defined by the presence of all four of the conserved BH domains (Figure 1B) [4]. The structures of both Bax and Bak are very similar to that of their pro-survival relatives. Both adopt a helical bundle defined by the same α-helical arrangement. The structure of Bax, determined by NMR, most resembles that of Bcl-w, whereby its α9 helix binds into its own hydrophobic groove, burying hydrophobic residues that would have otherwise been exposed (Figure 1F) [27]. This feature is thought to contribute to the cytosolic localization of this protein, which following a death stimulus translocates to the mitochondria by a mechanism that is still not well understood. In the case of Bak, the key difference between its structure and that of its pro-survival relatives, is its significantly constricted hydrophobic groove, which may contribute to the inhibition of ligand binding [28, 29]. One crystal structure of Bak also showed that it forms a zinc-dependent homodimer [28], where zinc plays an inhibitory role on its pro-apoptotic function. However, in a more recent study by Dewson *et al*., mutation of the proposed zinc-binding site had little effect on its activity [30].

### The pro-apoptotic BH3-only proteins

As their name suggests, the BH3-only proteins only possess homology with other Bcl-2 proteins in their BH3-domain. Most of these proteins (such as Bim, Bad and Bmf) are largely intrinsically unstructured proteins in the absence of their binding partners [25]. The only three-dimensional structure of a BH3-only protein solved to date is that of Bid (Figure 1G) [31, 32]. This structure closely resembles the fold of a multi-domain Bcl-2 protein (Figure 1C) but with some key differences, such as a much shallower hydrophobic groove that is typical of a pro-survival Bcl-2 protein. By using spectroscopic and sequence analyses, it has been predicted that in addition to Bid, the only other BH3-only protein that possibly possesses a defined structure is Bik [25], though this structure has yet to be solved.

### BH3 domains from BH3-only proteins in complex with pro-survival proteins

All current models of apoptotic regulation highlight the importance of interactions between pro-survival Bcl-2 proteins and pro-apoptotic BH3-only proteins. These interactions are mediated by the BH3 domain on the pro-apoptotic molecule. We now have numerous structures of these complexes with all pro-survival proteins, except Bcl-w, and of a variety of BH3 domains from BH3-only proteins including Bim, Puma, Bad, Noxa, Bid and Bmf (typically using synthetic BH3 domain peptides of ∼16-26 residues in length) [33–37] (Figure 1H). These complexes all demonstrate a similar mode of interaction: each of the four conserved hydrophobic residues on the helical BH3 domain project into hydrophobic pockets along the length of the conserved binding groove on the pro-survival protein. The conserved aspartic acid residue in the BH3 domain also forms a salt bridge with the conserved arginine residue in the BH1 domain found on all mammalian pro-survival proteins (i.e in the NWG**R** motif), and biochemical studies have shown this interaction is important for BH3 domain binding and biological activity. Additional interactions (hydrogen bonds, salt bridges) are also formed by some of the non-conserved residues along the BH3 domain with residues lining the groove.

Complexes of the same pro-survival protein bound to multiple BH3 ligands have demonstrated a degree of plasticity in the binding groove of some pro-survival proteins [35–40]. In Bcl-x_L_, for example, this usually involves helices α3 and α4, with α3 in particular seen to range from being highly helical in some structures to essentially unstructured, depending on the ligand [35, 36, 38–42]. (Figure 1I). Similar plasticity has also been noted for A1 (the mouse orthologue of Bfl-1) [37]. These differences in the binding groove results in alterations in the network of interactions that occur, particularly between the non-conserved residues in the BH3 domains and the residues lining the grooves. This is believed to be a determinant of the selectivity patterns apparent in BH3 domain:pro-survival protein interactions [37].

A different type of structural plasticity was observed in CED-9 when it binds to a peptide corresponding to the BH3 domain of the *C. elegans* BH3-only protein, Egl-1. Although Egl-1 BH3 engages the CED-9 ligand-binding groove similarly to the way mammalian BH3 sequences engage their pro-survival binding partners, a large movement of the unstructured loop region between helices α4 and α5 was noted when the liganded and apo structures are compared [43] (Figure 1J). This conformational change is required for downstream activation of the pathway, as discussed below.

Several groups, including us, have explored the idea of using BH3 peptides incorporating non-natural amino acids as a means of stabilizing them against proteolysis for therapeutic applications [39, 44–49]. Structures we have solved of BH3 peptides incorporating β-amino acids show similar binding modes to those of natural BH3 domains consisting of α-amino acids, even when the β-amino acids have non-native cyclic side chains [39, 44, 45]. Strategies to lock BH3 domains into a helical conformation using hydrocarbon staples have also been successful. A structure of one of these bound to Mcl-1 also suggests an essentially canonical binding mode [49], although additional interactions involving the staple itself were observed, and perhaps contribute to the high affinity of that ligand.

### BH3 domains from Bax/Bak in complex with pro-survival proteins

In addition to binding BH3-only proteins, pro-survival proteins also inhibit apoptosis by engaging Bax and Bak in their “activated” conformation. As with binding to BH3-only proteins, these interactions also involve the BH3 domains from the pro-apoptotic Bax/Bak molecules. At present our structural knowledge of these interactions is still limited to peptides of the Bax and Bak BH3 domains bound to various mammalian pro-survival proteins including Bcl-x_L_ [40, 50], Bcl-2 [51] Mcl-1 [50], as well as to a pro-survival protein from schistosomes [24]. These structures all show that they engage the same hydrophobic cleft that the BH3 domains from BH3-only proteins bind into, and that their mode of binding is very similar. Although these structures are limited to just the BH3 domains, they do provide some insights into the nature of the conformational changes that must occur when Bak and Bax adopts its active form. For example, the structures of full-length Bax and Bak show that the conserved hydrophobic residues on their BH3 domains, essential for binding pro-survival proteins, pack into the core of the protein [18, 27, 29]. Hence for these to be able to bind a pro-survival protein (or indeed another Bax/Bak molecule to form a homo-oligomer), the BH3 domain must become everted from the rest of the structure. Furthermore, our recent structures of the Bax BH3 domains bound to Bcl-x_L_ and Mcl-1 also indicate that this conformational change involves the α2 and α3 helices, which normally subtend an angle of nearly 90 degrees to one other in the apo form, but essentially becomes a contiguous helix upon pro-survival protein binding [50]. Hence, although these structures only represent a fragment of the entire Bax/Bak molecule, the information they contain extends somewhat beyond the binding interface.

### BH3 domains from BH3-only proteins in complex with Bax/Bak

The binding of BH3-only proteins to their pro-survival protein counterparts is one mechanism by which Bax/Bak activation can occur. The alternate way is through the direct binding of “activator” BH3-only proteins to Bax and Bak. Whilst many structures of BH3-only proteins in complex with pro-survival proteins have been determined, the structures of BH3-only proteins in direct complex with either Bax or Bak have proven elusive up until recent years. Utilizing NMR spectroscopy together with biochemical analyses, a model by which Bax activation through direct binding of BH3-only proteins, namely Bim, has been proposed [52, 53]. A novel binding site formed by α1 and α6 residues on Bax, distinct from the canonical hydrophobic groove engaged by BH3-only proteins on pro-survival proteins, was identified. This site is also defined by a surface hydrophobic groove, which is notably shallower than the canonical site on the opposite face of the molecule. Binding of Bim to this site triggers a series of structural changes that involves the displacement of the α1/α2 loop into an “open” conformation exposing a well-characterized N-terminal epitope. The putative transmembrane α9-helix is then mobilized to allow for membrane insertion and the Bax BH3 domain in turn is exposed. The exposed Bax BH3 domain propagates the activation of other inactive Bax molecules in the same way that Bim activates Bax. These studies importantly suggest a novel regulatory binding site on Bax that had not previously been considered. It will be interesting to determine if the same binding site also exists on Bak.

### Small molecule “BH3-mimetics” in complex with pro-survival proteins

One of the most important outcomes of the structures of BH3 domain:pro-survival protein complexes has been the development of small-molecule drugs that can induce apoptosis in a manner similar to that of the BH3-only proteins (i.e. by engaging the ligand-binding groove on the pro-survival protein). The best studied of such so-called “BH3-mimetics” is ABT-737 which binds Bcl-x_L_, Bcl-2 and Bcl-w with high affinity and was discovered through screening of small molecule fragment libraries that bind Bcl-x_L_ using NMR (“SAR by NMR”) [54, 55]. Insights into how such fragments could be assembled to form a high-affinity ligand were provided by the structures of the peptidic BH3 domain:pro-survival protein complexes. High-resolution structures of ABT-737 (and derivatives) bound to Bcl-x_L_ show exactly how it engages the ligand-binding groove, and how it might be altered to change its binding profile [38, 56]. An orally available form of ABT-737, ABT-263, is now in clinical trials for treatment of various cancers including leukaemia, lymphoma and small-cell lung cancer [57], and a more selective compound, ABT-199, which targets only Bcl-2 with high affinity has also recently entered clinical trials. Numerous other BH3-mimetics have been described [58], though many of these have been shown to kill cells *via* mechanisms that do not require Bax and Bak [59] suggesting they are not *bona-fide* BH3-mimetics. For most of these there is also no structural data to support their mode-of-action.

### The Bcl-2 protein family - What is missing?

There still remain some significant gaps in the structural biology of the Bcl-2 family of proteins. As highlighted above, we do not yet have any structures of full-length BH3-only proteins bound to pro-survival proteins, though these are not likely to be very revealing beyond the BH3 peptide-bound structures currently available, as most BH3-only proteins (with the exception of Bid and Bik) are likely to be intrinsically unstructured. More interesting, however, will be structures of full-length Bax or Bak bound to pro-survival proteins. These are likely to be informative as they should reveal both the “activated” conformations of Bax and Bak, and also whether there are binding interfaces in addition to the BH3 domain, as suggested by recent studies [14]. Similarly, there are no structures of a Bax/Bak oligomer of any type. Some recent structures of Bcl-x_L_ and Bcl-w dimers have provided some clues as to how these oligomers might form [60, 61], though none reveal a BH3-in-groove dimer suggested by the recent convincing biochemical data for how Bax/Bak dimerizes [62, 63]. A higher-order oligomer of Bax/Bak would also be extremely interesting, as this would reveal how the damage to the mitochondrial outer membrane occurs. This is the critical, point-of-no-return step in the apoptotic cascade, and it is still unclear whether well-defined pores are formed by the Bax/Bak oligomers, or whether they create less well-defined aggregates that simply lead to MOM damage resulting in leakage of the contents of the mitochondrial intermembrane space into the cytosol. In addition, a structure of a BH3 “activator” sequence bound to Bax/Bak would be useful for a complete understanding of how Bax/Bak activation occurs. Due to the unstable nature of the BH3 “activator”:Bax complex, only a model of the complex was inferred from key distance information obtained from NMR spectroscopy, though this was validated through biochemical and cell-based assays. Finally, we still don't fully understand why, given the similarities between the three-dimensional structures of the pro-survival proteins and Bax/Bak, does one confer a cell's survival whereas the other promotes its demise?

## STRUCTURAL BIOLOGY OF THE APOPTOSOME

Apoptosis has been well characterized in a number of model organisms and there are significant differences in the architecture of the cell death pathways between them. Nevertheless, they are all similar in that they culminate in the activation of the caspases. These proteases are typically present in cells in an inactive zymogen form, allowing for the rapid onset of cell death when required [64–66]. In vertebrates and insects two levels of activation occurs – first an “initiator” caspase (Caspase-9 in humans, DRONC in insects) is activated, and this can then cleave and activate downstream “effector” caspases (Caspase-3/7 in humans, DRICE in flies). In *C. elegans* the system is simpler due to the presence of just a single executioner caspase, CED-3. The active form of caspases is an obligate dimer, though the zymogen (procaspase) form of initiators is monomeric [64–66]. Activation and maturation of executioner caspases involves their proteolysis into a large (∼20 kDa) α and small (∼10 kDa) β subunits which form a (α/β)_2_ holoenzyme. The large subunit contains the catalytic dyad whilst residues important for substrate binding are contributed by the small subunit. Numerous structures of caspases have been solved in different forms including zymogen, active, as well as bound to chemical and protein inhibitors (e.g. p35 and IAP domains), and have been extensively reviewed elsewhere [64]. In short, the catalytic domain, derived from a single procaspase has the same fold in all caspases and is composed of α twisted β-sheet sandwiched between two layers of α-helices.

Whilst effector caspases are dimeric and are activated following cleavage by initiator capases, the initiators are monomeric, and require an activation “platform” that allows for their dimerization and auto-activation. Two models have been proposed for initiator caspase activation. A proximity induced dimerization model whereby the platform serves to increase the local concentration above the dissociation constant for dimer formation [67, 68]. By contrast, the induced conformation model argues that a conformational change, induced by the caspase binding to the platform, is most important for activation [69–71].

Perhaps the most exciting development in our understanding of caspase activation has come from the determination of structures of these activation platforms, “apoptosomes”, that facilitate this initiation of the caspase cascade. Apoptosome assemblies are found in a range of organisms and involve related oligomeric molecules: Apaf-1 in humans [72], CED-4 in *C.elegans* [73] and DARK in insects [74], which all appear to assemble differently for caspase activation. These proteins all have a similar domain architecture involving an N-terminal caspase recruitment domain (CARD) that can form homotypic interactions with CARD domains that are also found on the initiator caspases. This N-terminal protein interaction domain distinguishes initiator from effector caspases, which do not possess one. The CARD is followed by a nucleotide-binding α/β fold, a small helical domain (HD1), a winged helix domain (WHD) and then a second helical domain (HD2). Apaf-1 and DARK also have C-terminal regions that include WD40 domains. These molecules are known (together with the “R proteins” found in plants) as NB-ARC's (**n**ucleotide **b**inding, **A**paf-1, **R** proteins and CED-4) and belong to the AAA+ superfamily of ATPases. In the last five years, significant progress has been made into determining the structures of these death assemblies, providing critical insights into the mechanism by which apoptosis is executed in different organisms. Below we review key features of each of these structures, and highlight what we still do not yet fully understand about their mechanisms of action.

### The mamalian apoptosome

Unlike in the nematode (see below), activation of caspases in mammals requires cytochrome *c*, which is released from the mitochondria following Bax/Bak oligomerization. Cytochrome *c* is required for activation of Apaf-1 [72], which exists in the cytosol in an inactive or so-called “closed” conformation. It has been known for some time that switching to the “open” conformation following cytochrome *c* binding also involves exchange of a nucleoside diphosphate (ADP/dADP) for a nucleoside triphosphate (ATP/dATP) [75], though the exact roles of both cytochrome *c* and nucleotide in Apaf-1 activation have been obscure. Apaf-1 (like CED-4) contains a AAA+ ATPase domain (with Walker A and Walker B boxes for binding ATP and Mg2^+^), and has been reported to possess low level ATPase activity, though non-hydrolysable analogues of ATP can induce apoptosome formation [76], hence it was uncertain whether chemical energy from nucleotide hydrolysis was required for the assembly process.

**Figure 2:**
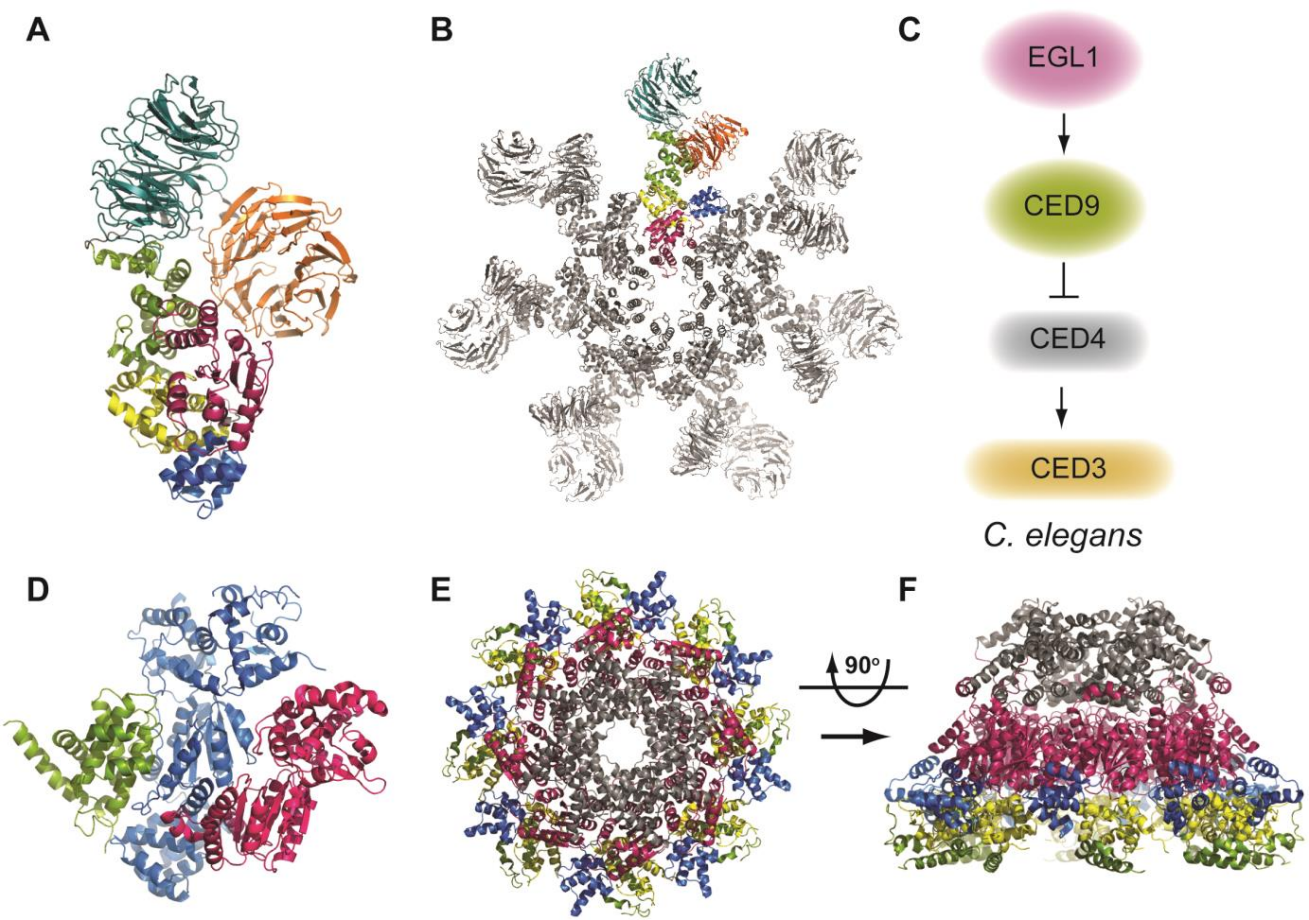
**A)** Crystal structure of Apaf-1 (PDB: 3SFZ). The domains are colored as follows: NBD (pink), HD1 (blue), WHD (yellow), HD2 (green), WD1 (seven blade propellor, orange), WD2 (eight blade propellor, teal). **B)** Cryo-EM structure of the DARK apoptosome (PDB: 3IZ8) shows an octameric platform (color coding as in part A); **C)** Schematic of the apoptotic cell death pathway in *C.elegans*. **D)** Crystal structure (PDB: 2A5Y) of CED-9 (green) bound to a CED-4 dimer (subunits in blue and pink, **E)** Top and **F)** side views of the crystal structure of the octameric CED-4 apoptosome (PDB: 3LQQ; CARD: grey, α/β-fold: pink, HD1: blue, WHD: yellow, HD2: green).

In 2005, a crystal structure of Apaf-1 bound to ADP was determined, though this lacked the C-terminal WD40 domains [77, 78]. Nevertheless, the location of the ADP molecule and the network of bridging interactions it forms suggested it could serve to lock the structure in an inactive conformation. Very recently, however, a crystal structure of full-length murine Apaf-1 was determined (Figure 2A) though, in this structure, electron density for the CARD domain was not visible (probably due to the high ionic strength of the crystallization buffer which disrupts charged interactions holding it in place and rendering it highly flexible) [79]. Nevertheless, a possible mechanism by which Apaf-1 is held in the closed conformation was apparent, and by comparison with a the cryo-electron microscopy (EM) model of the holo-apoptosome (Figure 2B) which represents the “open” conformation [80, 81], it was possible to assess the structural changes that probably occur following binding of cytochrome *c*.

In the structure, the WD40 repeats form tandem seven and eight blade β-propellers (as also predicted from a previous cryo-EM study) [80, 81] and these sit on top of the nucleotide binding and oligomerization domain. In their model, the authors propose that binding of cytochrome *c* between the β-propellers, causes one propeller (WD1) to swing out from its resting position, releasing the attachment of the nucleotide binding domain (NBD) and helical domain 1 (HD1) to the WD40 repeat regulatory domain. This movement of NBD1 and HD1 exposes the contact area for oligomerization and also relocates the CARD, allowing the apoptosome to assemble as a heptameric wheel with the CARDs arranged in a central hub. The mechanism they propose requires no chemical energy from nucleotide hydrolysis during the opening process, though exchange of ADP for ATP (which occurs in monomeric Apaf-1) is probably required as the γ-phosphate on the ATP is proposed to form a salt-bridge with Arg265, and this is important for the movement of the NDB-HD1 sub-domains.

As described above, cryo-EM studies provided important complementary data to the higher resolution crystallographic analysis of Apaf-1, and enabled insight into how it adopts its activated, oligomeric conformation. Associated biochemical analyses have also been very informative for providing a model of how caspase-9 and the Apaf-1 heptameric platform interact and function to activate caspase-3 [81]. This model shows that the CARD's of Apaf-1 and caspase-9 associate in a disk-like structure that sits above the platform. These CARDs are tethered by flexible linkers joining them to the rest of the protein, and these linkers are of a critical length for optimal activation. The CARD-disk appears to be located off-centre relative to the central hub of the platform. It is suggested that this asymmetry limits the number of procaspase-9 catalytic domain binding sites on the central hub, and consequently, that the holo-apotosome may only contain a single *active* procaspase-9, consistent with earlier biochemical studies that had also shown a stoichimetry of just 1 or 2 caspase-9 molecules per apoptosome, rather than 1 procaspase-9 per Apaf-1 molecule [82]. The binding of a single procaspase-9 molecule argues against the proximity-induced model of caspase-9 activation, and is consistent with the induced conformation model. Elegant biochemical studies from the Bratton group provide important additional insights into the dynamic nature of procaspase-9 interactions with the apoptosome and argue for a proteolytic-based molecular timer paradigm of caspase activation which is dictated, in part, by the differential affinities they report of procaspase-9 and the cleaved enzyme for Apaf-1 [82, 83]. In the structure, it also seems that procaspase-9 and caspase-3 have overlapping binding sites, and due to the asymmetry in the disk, multiple caspase-3 dimers could bind together with a singly bound procaspase-9 catalytic domain.

### Mammalian apoptosome - What is missing?

As described above, we now have available a combination of high-resolution structures for Apaf-1 (closed form) and low-resolution structures of the assembled apoptosome. Obviously, further higher resolution structures would aid in resolving the precise mechanism by which caspase activation occurs in mammals. For example, the open form of Apaf-1 could be useful for deciphering the role of nucleotide hydrolysis in apoptosome formation. Moreover, any structures of apoptosome complexes with caspases bound could resolve exactly how the zymogen forms are processed, though technical issues associated with producing all intermediate complexes, combined with the apparently dynamic and complex nature of activation steps, may restrict whether the entire process can be captured at high resolution.

### C. elegans apoptosome assembly and caspase activation

The cell death pathway that is perhaps best understood is that in *C.elegans* as we now have crystal structures of each event leading to the activation of the caspase CED-3. This pathway (Figure 2C) is fundamentally different to the intrinsic apoptosis pathway in humans as there is no Bax/Bak-like molecule required for the release of cytochrome *c* to switch the activation platform from its autoinhibited to activated state (as occurs with Apaf-1). Instead, the worm Bcl-2-like protein CED-9 inhibits cell death by directly engaging CED-4 (the Apaf-1 orthologue) at the mitochondria. In response to some death stimulus, the BH3-only protein Egl-1 is transcriptionally upregulated and binds to CED-9. This allows for the release of CED-4 into the cytosol where it can engage CED-3, leading to its activation and the eventual demise of the cell. Structural studies have now elucidated the mechanisms involved in the release of CED-4 by Egl-1 and the assembly of the apoptosome that allows for CED-3 activation.

A significant breakthrough in our understanding of how cell death is activated in nematode worms was achieved when the CED-4:CED-9 complex structure was determined [84, 85] (Figure 2D). Unexpectedly, this structure, together with associated biochemical studies, showed CED-4 forms an asymmetric dimer that is engaged with a single CED-9 molecule. This interaction involves a different interface on CED-9 from that involved in BH3 ligand binding. As described above, comparison of the apo-CED-9 structure with the BH3-bound CED-9 structure showed that, upon ligand binding, a significant conformational change occurs in the unstructured loop between helices α4-α5 which is close to the interface with one of the CED-4 molecules (Figure 1J). As such, movement of this loop disrupts the interaction with CED-4, and releases it into the cytosol where it can then oligomerize.

The second key study which revealed the structural basis of the *C.elegans* apoptsosome assembly was published in 2010 and provided further surprises regarding the mechanism of caspase activation. This structure showed that the assembled apoptosome is an octamer consisting of a tetramer of the asymmetric dimer that is bound by CED-9 [86] (Figure 2E, F). As such, no major conformational change is associated with the CED-4 apoptosome assembly (unlike the Apaf-1 apoptsosome). The surprise here was that the octamer forms a funnel like structure with the CARDs located toward the narrow end and the other domains extending essentially linearly towards the wider end (Figure 2F). A principle determinant of this apoptosome assembly is the interactions involving the α/β domain, both within a CED-4 molecule and between adjacent CED-4 dimers. Further biochemical and structural studies using cryo-electron microscopy revealed that, unexpectedly, each apoptosome only binds to two CED-3 molecules within the space, or “hutch”, inside the funnel. This localization of just two CED-3 molecules within this hutch apparently facilitates their dimerization, enhancing its proteolytic activity.

### C. elegans apoptosome - What is missing?

Whilst combined these studies provide unparalleled insight into apoptosome formation and caspase activation, there remain some unanswered questions. These primarily relate to how the CED-3 zymogen binds to, and is activated by, the apoptosome. The studies reported used purified recombinant CED-3 without its CARD domain due to solubility issues associated with producing the unprocessed form. Consequently, the role for the CED-3 CARD and the nature of the events involving the unprocessed form of CED-3 engaging the CED-4 CARD domains (which are located on top of the funnel), then adopting the active dimer conformation within the hatch are still to be determined.

### The fly apoptosome

The Drosophila apoptososme consists of the Apaf-1 orthologue DARK which assembles into a platform necessary for the activation of the initiator caspase DRONC, and downstream activation of the executioner procaspase, DRICE. As in mammals, DARK possesses a regulatory domain consisting of WD40 repeats, though mounting evidence suggests that cytochrome *c* is not required for DARK assembly or DRONC activation.

All of our knowledge of the Drosophila apoptosome structure has been obtained from cryo-EM studies [87–89]. An earlier study had suggested that the Drosophila apoptosome could be a double-ringed structure [88], though a more recent study showed this was probably a concentration-dependent phenomenon, and that a single-ringed structure (as seen in the human apoptosome) is the more likely physiologically relevant form [89].

In several studies, including the most recent report of a 6.9Å structure, the DARK single (and double) ring(s) are shown to consist of eight subunits (compared to seven in the Apaf-1 assembly) with the hub of the wheel-like complex comprising the NBD's [89] Unlike in Apaf-1 and CED-4, the CARDs are positioned on top of the central hub creating a crown-like arrangement. Interestingly, there is a clear path for dATP exchange in the Drosophila apoptosome, due to a much shorter HD1-WHD linker compared to that in Apaf-1. *In vitro*, dATP exchange appears to provide the driving force for DARK platform assembly as cytochrome *c* is not required, and although an alternative activating ligand cannot be entirely ruled out, assembly of a DARK-DRONC apoptosome capable of cleaving DRICE (without cytochrome *c*) suggests that such a ligand is probably not required.

### Fly apoptosome - What is missing?

Our knowledge of the fly apoptosome comes entirely through relatively low-resolution cryo-EM studies. Hence, higher resolution crystal structures are required. Structures of the assembled apoptosome that include DRONC will also be important, as modelling based on the current best structure suggests that the DRONC CARD can engage the DARK CARD's, but at higher radius a clash likely occurs with the seven-blade propellor, suggesting some structural re-arrangements occur during caspase activation. The nature of such structural changes is lacking and will be required for a complete understanding of caspase activation in the fly.

## SUMMARY AND OUTLOOK

Structural studies have been central to our understanding of how apoptosis is regulated. Indeed, these structures have sometimes revealed surprises that would not have been apparent if we relied only on biochemical and biological studies for informing how the process is controlled. It is particularly interesting that whilst all of the apoptotic cell death pathways studied to date possess similar components with very similar three-dimensional structures, these all engage each other in different ways, resulting in vastly different mechanisms for the activation of the caspases, the ultimate event in all apoptosis pathways. As indicated above, some significant gaps in the structural biology of this process still exist, though the available structures have enabled drugs such as ABT-263 to be developed. Hopefully, future advances that close the remaining gaps will not only provide us with a more complete picture of apoptotic regulation (and evolution) at high-resolution, but perhaps provide further clues as to how new drugs could be developed to intervene in cells where apoptosis is dysregulated.
